# Polymorphism in the TNF-*α*(−863) locus associated with reduced risk of primary open angle glaucoma

**Published:** 2012-03-31

**Authors:** Chun-Yuan Wang, Ying-Cheng Shen, Li-Chen Wei, Keng-Hung Lin, Shih-Chao Feng, Yi-Yin Yang, Chun-Hung Chiu, Hin-Yeung Tsai

**Affiliations:** 1 Department of Ophthalmology, Taichung Veterans General Hospital, National Yang-Ming University, Taichung, Taiwan, Republic of China; 2 Hung Kuang University, Taichung, Taiwan, Republic of China; 3 Central Taiwan University of Science and Technology, Taiwan, Republic of China; 4 Jen-Teh Junior College of Medicine, Nursing and Management, Taiwan, Republic of China; 5 Chung Hwa University of Medical Technology, Tainan, Taiwan, Republic of China

## Abstract

**Purpose:**

Tumor necrosis factor-α (TNF-α), an important proinflammatory cytokine, exerts a variety of physiologic and pathogenic effects that lead to tissue destruction. Recent laboratory evidence indicates that TNF-α have either protective or adverse effects on primary open angle glaucoma (POAG). Inheritance of the TNF-α (−863) C allele has been associated with an elevated risk of Alzheimer disease. The neuronal injuries associated with Alzheimer disease have several similarities with the optic nerve changes often seen with POAG. In this study we investigated the possible association between the TNF-α (−863) polymorphism and the development of POAG.

**Methods:**

A total of 234 patients with POAG were recruited and compared with 230 healthy controls in a Chinese population. Sequence-specific primers with 3′ end mismatches were used to identify the presence of specific allelic variants by polymerase chain reaction (PCR) amplification. Patients and controls were genotyped for the A/C polymorphism at position −863 of the TNF-α gene promoter region.

**Results:**

The frequency of the TNF-α (−863)A allele (22% versus 30%, respectively; p=0.007) and the carriers of the TNF-α (−863)A allele (37% versus48%; p=0.017, OR 0.63, 95% CI 0.44–0.92) were lower in POAG patients compared with those in controls. There is a reduced risk of POAG associated with homozygosity for the TNF-α (−863)A allele (AA genotype) compared with that in the control population (AA genotype; 7% versus 11%, respectively, p=0.037; OR 0.5, 95% CI 0.26–0.98).

**Conclusions:**

The TNF-α (−863)A allele polymorphism may be a protective factor in the development of POAG.

## Introduction

Glaucoma is a progressive optic neuropathy characterized by degeneration of retinal ganglion cells, cupping of the optic nerve heads and visual field defects often related to elevated intraocular pressure. Glaucoma affects 70 million people worldwide, and constitutes the second largest cause of bilateral blindness in the world [1]. Primary open angle glaucoma (POAG) is a multifactorial neurodegenerative disease. Both genetic and environmental factors are thought to contribute to the pathophysiology of the disease.

Glaucoma is a complex clinical trait and its inheritance has been shown to follow both Mendelian and non-Mendelian models [2]. At least eight loci of genes have been found to be associated with POAG, such as myocilin (*MYOC*), optineurin (*OPTN*) and WD repeat domain 36 (*WDR36*) [3-6]. Mutation in the optineurin gene was initially reported in 16.7% of families with hereditary POAG, with most of them having normal tension glaucoma [4]. Cytochrome P450, family 1, subfamily B, polypeptide 1(*CYP1B1*) and latent-transforming growth factor beta-binding protein 2 (*LTBP2*) mutations are more frequent in congenital and developmental glaucoma [7-9]. Other genetic factors, e.g., optic atrophy 1 (*OPA1*), apolipoprotein E, E-cadherin, neurotrophin-4 (*NTF-4*) and opticin (*OPTC*), have been reported to elevate the risk of retinal degeneration, a characteristic of glaucoma [10-14]. However, these genes can not account for the overall inheritance of susceptibility to POAG pathogenesis. Other associations involved in the development of POAG should also be investigated. Genetic association studies aimed at defining susceptibility to POAG may provide important insights into the pathogenesis of POAG.

There is evidence to support the hypothesis that the immune system plays a potential pathogenic role in glaucomatous optic nerve degeneration [15,16]. Balancing the benefit of protective immunity and the risk of inducing an autoimmune neurodegenerative disease is critical, as the effect of such immune regulation may be either neuroprotective or neurodestructive [17]. T-cell mediated immune response may initially be beneficial in limiting neurodegeneration. However, a failure to properly control aberrant, stress-induced immune response likely converts the protective immunity to an autoimmune neurodegenerative process that can facilitate the progression of neurodegeneration in glaucoma patients [17,18]. For example, increased numbers of auto-antibodies were found in the serum of glaucoma patients, and such auto-antibodies include those with specificities to heat shock proteins such as heat shock protein 60 (HSP60) and HSP27, alpha crystallins and HSP70 [19,20]. Tumor necrosis factor-α (TNF-α) is a contributing factor in the neurodegeneration in glaucoma [21-24].The association between the degree of immune responses and the development of glaucomatous optic nerve degeneration may be fluid, and in certain cases may result in retinal ganglion cell death through an aberrant immune signaling process.

A C/A polymorphism at position −863 of the *TNF-α* promoter region has been reported to be associated with Alzheimer disease [25,26]. There is evidence that the TNF*-α* protein may act to promote the development of β-amyloid deposits [27]. Similar evidence also indicates that there is β-amyloid build-up in retinal ganglion cells in rats with experimental glaucoma [28,29]. Tamura et al. [30] have shown a high frequency of POAG in patients with Alzheimer disease. In this regard, glaucoma may be viewed as a chronic neurodegenerative disease similar to Alzheimer disease, and a slow build up of β-amyloid in ganglion cells may eventually trigger cell death and optic nerve axon loss.

Recent studies have shown that ischemic or pressure-loaded glial cells produce TNF-α, which results in oligodendrocytes death and the subsequent apoptosis of retinal ganglion cells [31]. Tezel et al. [21] found that TNF-α or TNF-α receptor-1 (TNF-R1) are raised in the retina of glaucomatous eyes compared with levels in normal eyes, suggesting that cell death mediated by TNF-α is a contributing factor in neurodegeneration in glaucoma. Balaiya et al. [32] found TNF-α levels in aqueous humor were elevated in patient with POAG. Other evidence showed that anti- TNF-α antibodies can prevent death of retinal ganglion cells (RGCs) by reducing ocular hypertension, which indicates that reducing the expression of TNF-α would be beneficial in treating glaucoma [33].

To date, several publications have evaluated the association between *TNF-α* promoter polymorphisms and glaucoma risk [34-39]. However, the results remain inconclusive. Inheritance of the *TNF-α* (−863) C allele has been associated with an elevated risk of Alzheimer disease. A C/A polymorphism at position −863 of *TNF-α* promoter region is considered to be an important enhancer of transcriptional activation associated with elevated levels of TNF-α. Given the potential similarities in cellular events leading to neurodegeneration between Alzheimer disease and glaucoma, we hypothesized that *TNF-α* (−863) polymorphism may be a genetic factor predisposing affected individuals to glaucoma due to its effect on TNF-α protein expression. Thus, we investigated the distribution of the *TNF-α* (−863) polymorphism in patients with POAG and compared them with that in a healthy control population.

## Methods

### Study subjects

Subjects were recruited from the outpatient clinic at the Department of Ophthalmology of Taichung Veterans General Hospital, Taiwan from January 2009 to July 2011. POAG patients were invited to participate in the study when they came to the clinic for a previously scheduled visit, and were enrolled after informed consent was obtained. Normal control subjects were recruited during their visits to the outpatient clinic for various other reasons. In total, 234 patients with POAG and 230 normal controls were enrolled (Table 1). The mean age was 67 years in the POAG group (range 25–78) and 67 years in the control group (range 44–79). There was no difference in age between the control and POAG groups (p>0.05). Patients were followed up for between 1 and 10 years (with a mean of 5 years). Twenty-one of the patients had received trabeculectomy and four of the 21 patients had received trabeculectomy twice from different sites. Of the POAG patients, 217 were prescribed antiglaucoma eyedrops. All medical treatment primarily included topical beta-blockers and prostaglandin. Each patient used an average of 1.4 types of antiglaucoma drugs. Seventeen patients did not need medication to control intraocular pressure (IOP) after trabeculectomy.

**Table 1:** Baseline characteristics of patients and controls.

** Parameter**	**POAG (n=234)**	**Control (n=230)**
Mean age (years)	67	67
Range (years)	25–78	44–79
Male (%)	52	53
Mean IOP (±SD; mmHg)	26±3.5	17±2.1
Mean vertical cup-disc ratio (±SD)	0.84±0.13	0.4±0.10

IOP: intraocular pressure; SD: standard deviation.

All participants received comprehensive ophthalmologic examinations, including visual acuity testing with refraction, corneal thickness, IOP measurement, Humphrey 30–2, slit lamp examination, Stratus optical coherence tomography (Carl Zeiss Meditec, Inc., Dublin, CA), and dilated slit lamp stereo biomicroscopy.

Comprehensive ophthalmologic history and longitudinal follow-up data were also obtained for each individual. The definition of POAG included characteristic arcuate, Bjerrum, Seidel and/or paracentral scotoma and/or nasal step on Humphrey 30–2 with reference to Anderson’s criteria for minimal abnormality in glaucoma [40], and corresponding cupping of optic nerve heads and/or nerve fiber layer defects on stereo biomicroscopy, open drainage angles on gonioscopy, and the absence of a secondary cause of glaucomatous optic neuropathy, such as previous trauma, a period of steroid administration, or uveitis. The POAG cases had an elevated IOP of >21 mmHg on diurnal testing. Patients with a history of inflammation, ocular hypertension, congenital glaucoma or normal tension glaucoma were excluded.

Unrelated control subjects were recruited from those attending the clinic for senile cataract, floaters, refractive errors, or itchy eye. All normal control subjects had no systemic disease and no family history of glaucoma. Glaucoma in these patients was excluded using the same diagnostic criteria used for the POAG patients after the same ophthalmic examination procedure. If there was any doubt whether the subject had NTG or POAG, the subject was excluded. The study was performed with the approval of the Human Study Committee of Taichung Veterans General Hospital. Written informed consent was obtained from all study subjects before enrollment.

### DNA preparation and genotyping

Blood samples were collected from each subject (5 ml) and genomic DNA was isolated using the Quiagen QiaAmp Blood mini kit (Quiagen, Valencia, CA). Polymorphisms were determined using sequence-specific primers (SSPs). PCR-SSP uses SSPs with 3′ end mismatches and identifies the presence of specific allelic variants through PCR amplification. For the polymorphisms in the *TNF-α* (−863), we used the primer sequences and primer mixtures previously described by Fanning et al. [41] and Grutters et al. [42]. The promoter polymorphism -863(C/A) was identified with the sequence-specific forward primers 5’-CGA GTA TGG GGA CCC CCC-3' and 5’-GAG TAT GGG GAC CCC CA-3' in combination with the consensus reverse primers 5’-CCG GGA ATT CAC AGA CCC C-3' at a final concentration of 20 ng/μl, with an expected PCR product size of 263 bp.

PCR products were run on ethidium bromide-stained 2% agarose gels. We sequenced the 5′ regulatory region (containing the promoter) of *TNF-α* in selected individuals with each genotype (CC, CA, and AA) to confirm the validity of PCR assay. Complete matching results in PCR assay and DNA sequence were obtained. To ensure accuracy, each test was performed three times for each sample.

### Statistical analysis

Genotype and allele frequencies between the control and POAG groups were compared using the χ^2^ test, and Fisher’s exact test was used if any expected frequency was lower than 5. Age and gender were compared between the control and POAG groups using the Mann–Whitney U test and χ^2^ test, respectively. Odds ratios were computed to assess the strength of associations between the presence of each genotype and the clinical diagnosis of POAG. A p-value of less than 0.05 was considered statistically significant. All statistical analyses were performed using SPSS 13.0 (SPSS Inc., Chicago, IL).

## Results

### *TNF-α* (−863) A association with reduced risk of POAG

The distributions of *TNF-α* (−863) genotypes and alleles are shown in Table 1. In POAG patients, the genotype *TNF-α* (−863)A/A was less frequent than in controls (7% versus 11%; p=0.037; Table 2). The frequency of the *TNF-α* (−863)A allele was significantly decreased in POAG group (22% versus30%, p=0.007). In comparison with controls there were significantly fewer carriers of the *TNF-α* (−863) A allele among patients with POAG (37% versus 48%; p=0.017), with an odds ratio of 0.63 (95% CI: 0.44–0.92; Table 2). The distribution of genotypes in the population of patients and controls was consistent with the Hardy–Weinberg equilibrium, with no significant detectable differences between expected and observed numbers.

**Table 2:** Genotype and allele frequencies of TNF-α (−863).

**TNF-α(-863) polymorphism**	**POAG (%; n=234)**	**Control (%; n=230)**	**Odds ratio (95% CI)**	**p-value**
**Genotype**				
C/C	148 (63%)	120 (52%)	1	NA
C/A	70 (30%)	84 (37%)	0.68 (0.45, 1.01)	0.067
A/A	16 (7%)	26 (11%)	0.5 (0.26, 0.98)	0.037
**Allele**				
C	366 (78%)	324 (70%)		
A	102 (22%)	136 (30%)	0.66 (0.49, 0.89)	0.007
**A allele carriage**				
AA+CA	86 (37%)	110 (48%)	0.63 (0.44, 0.92)	0.017
CC	148 (63%)	120 (52%)		

Distribution of genotypes and allelic frequencies in primary open angle glaucoma (POAG) and controls. In POAG patients, the genotype *TNF-α* (−863)A/A was less frequent than in controls (7% versus 11%; p=0.037). The frequency of the *TNF-α* (−863)A allele was significantly decreased in POAG group (22% versus 30%, p=0.007).There were significantly lower carriers of the *TNF-α* (−863)A allele among patients with POAG than among controls (37% versus 48%; p=0.017), the odds ratio was 0.63 (95% CI: 0.44–0.92).

## Discussion

In this study, the genotype *TNF-α* (−863)AA was less frequent in POAG patients than in controls (7% versus 11%; p=0.037). The frequency of the *TNF-α* (−863)A allele was significantly decreased in POAG group (22% versus30%, p=0.007). There were significantly fewer carriers (AA or CA) of the *TNF-α* (−863) A allele among patients with POAG than control (37% versus 48%). Our results indicate that *TNF-α* (−863) A allele polymorphism may be a protective factor in the development of POAG. Inheritance of the *TNF-α* (−863) C allele has been associated with elevated risk of developing Alzheimer disease [25,26]. There is evidence that the TNF-α protein may act to promote the development of β-amyloid deposits in Alzheimer patients [27], and McKinnon and colleagues [28,29] have demonstrated evidence of build up of β-amyloid in retinal ganglion cells in a rat glaucoma model. The death of retinal ganglion cells in glaucoma involving chronic β-amyloid neurotoxicity may mimic Alzheimer disease at the molecular level. These data point to a potential overlap between the degenerative pathways underlying POAG and Alzheimer disease.

TNF-α, a pleoprotic proinflammatory cytokine, is upregulated in several neurodegenerative disorders including multiple sclerosis, Parkinson disease, Alzheimer disease [43,44], multiple sclerosis [45], and in optic nerve microglia and astrocytes in glaucoma patients [46,47]. TNF-α has either protective or adverse effects on POAG. TNF-α is considered to exert a neuroprotective effect, because it activates the ubiquitous transcription factor, nuclear factor kappa B (NF-κB),through binding to the high affinity TNF receptor (TNF-R2). TNF-α can also serve as a neurodegenerative factor when it binds to the low affinity death receptor TNF-R1 and induces the mitochondria-mediated apoptotic pathway [48-52]. An increased expression of TNF-α can shift the balance toward TNF-R1 signaling, as seen in glaucoma, and thus promote retinal ganglion cell death. Increased TNF-α levels have been associated with a poor prognosis after trauma in the brain [53], whereas a decrease in TNF-α is known to reduce nerve damage [54].

The *TNF* gene lies within the human leukocyte antigen (*HLA*) class III region, located 250 kb centromeric to the *HLA-B* locus and 850 kb telomeric to the class II *HLA-DR* locus on chromosome 6p21.3. Recent studies have reported new promoter polymorphisms in the *TNF-α* gene, located at position −863, which may be responsible for transcriptional regulation of TNF-α protein production [55]. In *TNF-α*, the *TNF-α* (−863)A allele has also been linked with 31% lower transcriptional activity in reporter gene studies owing to reduced binding affinity of the transcription nuclear factor (NF-κB), especially the form p50-p50 [56,57]. The −863A allele has been reported to be associated with reduced circulating levels of TNF-α protein [56,58].

Several publications have evaluated the association between the *TNF* promoter polymorphisms and glaucoma risk [34-39]. Lin et al. [35] found that the *TNF-α* (−308)G/A polymorphism has been reported to be associated with POAG in a Chinese population; Razeghinejad et al. [38] revealed the importance of the *TNF-α* (−308)G/A polymorphism in the development of POAG and PEXG in Iranian patients. The *TNF-α* (−308)G/A polymorphism has been reported to be associated with PEXG in the Pakistani and Turkish population [36,39]. Funayama et al. [34] investigated sequence variation in *OPTN*, the expression of which is induced by TNF-α, and their association with polymorphisms in the promoter region of *TNF-α* at position −308,-857, and −863 in Japanese patients with POAG. However, no significant difference in genotype or allelic frequency was noted between patients and Japanese control for the three single-nucleotide polymorphisms of *TNF-α*. Our study showed that the *TNF-α* (−863)A allele is associated with POAG protection in a Chinese population. The genotype and allele frequency in our control population was different from that observed in Japanese populations [34]. The frequency of the *TNF-α* (−863)A allele were lower in Japanese populations compared with those in Chinese populations (13% versus 29%, respectively; p<0.05). In the Japanese study, the frequency of the AA genotype was as rare as 0.5%. In contrast, the frequency of the AA genotype at −863 was reported to be more than 10% in Chinese populations. These findings indicate Chinese and Japanese have a different genetic background at this site. This phenomenon may be due to ethnic differences, which may lead to controversial results.

In our study, we noted the *TNF-α* (−863)A allele polymorphism may be a protective factor in the development of POAG. Besides the *TNF-α* gene, previous studies have noted other cytokine genes (interleukin 1-α, *IL-1β*) and growth factor gene (insulin-like growth factor-II) polymorphisms are associated with an elevated risk of POAG [34,35,59-61]. Lin et al. [35] found that the *IL-1β* (+3953)T allele were significantly more common in POAG patients than in controls. In future studies, proteomic analyses may be useful for determining the exact roles of cytokines genes in the development of POAG.

Controversy exists regarding whether or not there is an absolute increased level of TNF protein in the vitreous or plasma that coincides with an increased level in retinal ganglion tissue among patients with POAG. The genotyping data obtained in the present study may now enable researchers to identify potential subpopulations within POAG patients, who we predict are likely to have increased circulating cytokines in the vitreous fluid. These data also further strengthen the current hypothesis that neuro-inflammation is a contributing component in the pathogenesis of POAG.

Elevated IOP is an important risk factor for glaucoma. Thus, an understanding of the genes that are involved in apoptosis, oxidative stress, mitochondrial dysfunction and regulating IOP should assist in the elucidation of the mechanisms of glaucoma. In our study, glaucoma features such as IOP and vertical cup-disk ratio were included. There were no significance between the *TNF-α* (−863) allele frequency and different level of IOP or stage of POAG. The association still needs further study.

The results of our genetic analysis showed that the *TNF-α* (−863) A allele is associated with POAG protection in a Chinese population. Further studies on POAG patients with different ethnicities are required to further define this association. The possibility that linkage disequilibrium between *TNF-α* gene polymorphisms and other genes on chromosome 6 is the cause of increased risk of POAG can not be excluded.

In conclusion, our results indicate that the *TNF-α* (−863) A allele polymorphism may be a protective factor in the development of POAG. The data indicate that therapeutic strategies designed to reduce TNF-protein production or activity might be a valuable treatment for POAG. Further insight into the mechanism underlying this relationship may suggest new strategies for reducing the intensity of the inflammatory response and attenuating the progression of POAG.
